# Evolution of species interactions determines microbial community productivity in new environments

**DOI:** 10.1038/ismej.2014.215

**Published:** 2014-11-11

**Authors:** Francesca Fiegna, Alejandra Moreno-Letelier, Thomas Bell, Timothy G Barraclough

**Affiliations:** 1 Department of Life Sciences, Imperial College London, Ascot, Berkshire, UK

## Abstract

Diversity generally increases ecosystem productivity over short timescales. Over longer timescales, both ecological and evolutionary responses to new environments could alter productivity and diversity–productivity relationships. In turn, diversity might affect how component species adapt to new conditions. We tested these ideas by culturing artificial microbial communities containing between 1 and 12 species in three different environments for ∼60 generations. The relationship between community yields and diversity became steeper over time in one environment. This occurred despite a general tendency for the separate yields of isolates of constituent species to be lower at the end if they had evolved in a more diverse community. Statistical comparisons of community and species yields showed that species interactions had evolved to be less negative over time, especially in more diverse communities. Diversity and evolution therefore interacted to enhance community productivity in a new environment.

## Introduction

**Figure 1:**
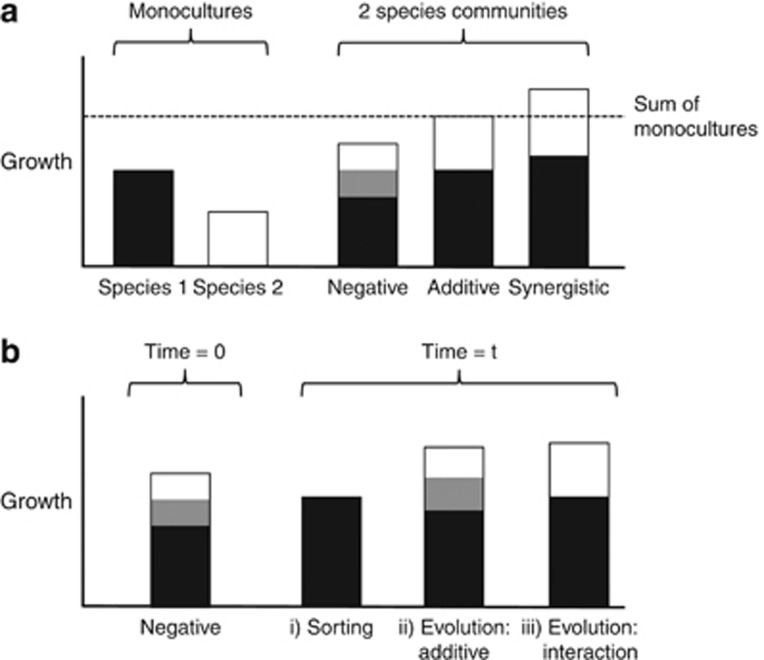
(**a**) Species interactions classified based on the relationship between community growth and the growth of constituent species in monoculture (Foster and Bell, 2012). Negative interactions, such as competition for shared resources, lead the community to grow worse than the sum of the species yields. Additive growth equal to the sum of monoculture growth (dashed line) is expected if species use non-overlapping resources and do not interact in other ways. *Synergistic* indicates that one or both species benefit so that combined growth is greater than additive. The frequency of different types will determine the shape of the relationship between community growth and species richness. Note that this classification focuses on net effects rather than mechanisms: for example, resource use, toxin production, signalling and interactions mediated by phage could all affect net interactions. (**b**) Community growth can change over time via three mechanisms, shown with an initially negative interaction as an example: (i) *Ecological sorting* occurs through the loss of one of the species, leading to lower community growth in this example; (ii) *Evolution* changes the growth of each species but their interaction (represented by the grey area) remains the same (which we call *additive* evolution; in this example growth of both species increase); (iii) *Evolution* alters the strength of the species *interaction* even if separate growths remain unchanged (in this example at time *t* there is no longer a negative interaction).

Diversity generally increases measures of ecosystem functioning; for example, biomass and productivity tend to be higher in experimental communities that contain more species (Bell *et al.,* 2005; Hooper *et al.,* 2005). This occurs because more diverse communities are more likely to both include highly productive species and use a wider range of resources as a result of niche differences among species (Loreau and Hector, 2001). The shape of the relationship depends on the types of species interactions (Figure 1; Connolly *et al.,* 2013). Diversity can also increase robustness of community functioning to environmental perturbations, for example, the productivity of experimental bacteria communities exposed to different abiotic conditions (Awasthi *et al.,* 2014). Most studies, however, have measured functioning over short timescales of a few generations. Understanding how ecological and evolutionary processes affect functioning over longer timescales is needed to predict, for example, how insect and plant communities respond to climate change or gut microbes to antibiotic treatments (Norberg *et al.,* 2001; Isbell *et al.,* 2011; Reich *et al.,* 2012).

Changes in functioning in a new environment will depend in part on ecological changes in the composition and relative abundance of species. Some species will grow better in new conditions than others, and poorly growing species might go extinct (Steudel *et al.,* 2012; Mori *et al.,* 2013). Functioning will then depend on the traits of the surviving species after ecological sorting rather than the original species complement. Changes in functioning might also depend on evolution of traits of constituent species in response to selection from new environmental conditions or from shifts in the surrounding biotic community. Evolution might improve ecosystem functioning. For example, evolution of enhanced growth of constituent species, on average, would increase community productivity. In addition, selection for greater specialisation and niche partitioning could increase the range of resource use and total growth of the community (Gravel *et al.,* 2011; Lawrence *et al.,* 2012). Alternatively, evolution might decrease productivity if some species evolve to monopolise resources, to inhibit other species via toxin production or in other ways destabilise the community (Bell, 2007).

Crucially, the extent of evolution might itself depend on diversity. Recent theory predicts that the average rate of evolution declines with the number of species in a community (Johansson, 2008; de Mazancourt *et al.,* 2008). This occurs either because the average population size decreases or because ecological interactions such as competition limit the opportunity for new phenotypes to evolve (Price and Kirkpatrick, 2009). Alternatively, greater diversity might increase evolution by increasing the strength of biotic interactions (Liow *et al.,* 2011) or amplifying the strength of selection caused by an environmental change (Osmond and de Mazancourt, 2013; Pekkonen *et al.,* 2013). The effects of evolution on functioning might also vary systematically with diversity. Loeuille (2010) showed that evolution of interaction strengths enhanced stability of theoretical Lotka–Volterra communities with <30 species but reduced stability in more diverse communities. Although the model did not consider productivity, similar effects could be envisaged for productivity.

Few studies have tested these ideas empirically. Selecting for increased specialisation of bacteria species led to steeper diversity–productivity relationship in community microcosms (Gravel *et al.,* 2011). However, the species evolved separately rather than in a community. Species interactions between five co-occurring bacterial species have been shown to stimulate divergence in resource use, leading to enhanced productivity of the entire community (Lawrence *et al.,* 2012). Yet, it remained unclear whether similar effects would be found with increasing numbers of species: coevolution might be constrained as diversity increases.

We investigated the effects of diversity and evolution on community productivity using artificial communities of bacteria isolated from pools among the roots of beech trees, called tree-holes. Previous work showed that overall productivity increases with diversity in this system (Bell *et al.,* 2005) and that evolution in simple communities can promote functioning (Lawrence *et al.,* 2012). Here, we used replicated microcosms of 1, 2, 3, 6 and 12 species to determine how evolution and productivity varies with increasing diversity under changed environmental conditions. Microcosms were cultured using serial transfer for ∼20 generations in standard laboratory conditions and then in three different environments for a further circa 60 generations: standard beech-leaf tea medium that mimics natural resources for these species; a lower pH5 environment, still grown on beech tea, chosen to reflect an acidity at the extreme of what is found in tree-holes and because pH is the main determinant of community diversity and composition in broad surveys (Griffiths *et al.,* 2011); and an alternative resource environment, namely spruce tea that has a higher carbon to nitrogen ratio and proportion of cellulose to lignin (Kalbitz *et al.,* 2006).

Changes in community productivity during the experiment could result because some species went extinct, because surviving species evolved to grow better (or worse) or because species evolved greater (or lesser) niche differences or by other means altered their interactions (Figure 1b). Each of these processes could affect the relationship between productivity and diversity. By comparing responses of monocultures and whole communities, we partitioned observed changes into those caused by ecological sorting, those due to additive changes in species yields and those due to evolution in the strength and nature of species interactions. Evolution of species interactions was the main contributor to changing productivity across ecosystems, especially in more diverse microcosms. We cannot infer the mechanism of species interactions based on data here. However, previous evidence on a single simplified community supports the hypothesis that changes result from changing resource use.

## Materials and methods

### Species and media

Samples of water were collected from a single tree-hole among the roots of a beech tree in Silwood Park, Berkshire, UK, in November 2008, and aliquots were spread on R2A agar plates (Oxoid Ltd, Basingstoke, UK). Twelve isolates were chosen that had different growth phenotypes on agar plates that would allow for their separation from mixed cultures at the end of the experiment. The 16S rDNA sequences showed that species belonged to six bacterial families and four phyla (Table 1; 16S sequences deposited in Genbank, accession numbers KJ598020–KJ598031; Supplementary Methods).

Beech tea medium was prepared by autoclaving 50 g of beech tree leaves in 500 ml of dH20, filtering it and diluting 32-fold in water. Spruce tea was prepared using 50 g of spruce needles in 500 ml water, and diluting it 32-fold. The media were supplemented with R2A agar ingredients (Reasoner *et al.,* 1979) to increase growth rates and thereby increase the number of generations during the experiment (Supplementary Methods). Beech tea and spruce tea media were initially pH 7.1 and 6.8, respectively, and were buffered to pH 7 and the beech tea for the pH5 treatment was buffered to pH5 by adding phosphate buffer (Supplementary Methods).

### Evolution experiment

Each species was cultured in monoculture, in a full mix of all 12 species, and in two alternative compositions in two-, three- and six-species mixtures in turn using a random partitioned design (Bell *et al.,* 2005, 2009). In the two-species mixtures, the species were subdivided into six random pairs. If two species with similar growth morphology were assigned together (for example, both had white colonies), one was swapped randomly with one species from another pair to maximise distinguishability. The procedure was repeated with different random pairings to obtain the second set of compositions for the two-species mixtures. An equivalent procedure was used to assign the three- and six-species mixtures. There were 37 different species compositions in total including monocultures and all communities (Supplementary Table S1).

To start the microcosms, species were inoculated at approximately the same optical density (OD) at 595 nm into 10 ml of medium in glass test tubes in the appropriate experimental mixtures. All assemblages were replicated thrice (3 × 37=111 microcosms). Microcosms were kept at room temperature and static. Alternating every 3 or 4 days, an aliquot (150 μl) was transferred to fresh medium to maintain microcosms in a state of active growth and cell doubling during the experiment. After 2 weeks in standard beech tea medium, aliquots from each microcosm were transferred into three separate tubes: one with standard beech tea, one with pH5 beech tea and one with spruce tea (3 × 111=333 microcosms). Transfers to fresh medium were performed alternating every 3 or 4 days maintaining the appropriate environmental treatment for each tube. Cultures were kept for further 5 weeks.

We measured the biomass yield of each microcosm during each serial transfer period as the maximum OD minus the starting OD. OD is a robust measure of biomass and scaling factors to convert from OD to g dry weight are fairly constant across organisms (Myers *et al.,* 2013). We considered biomass rather than cell densities because biomass is directly comparable across microcosms and better reflects productivity (Awasthi *et al.,* 2014). Changes in biomass during the experiment could in principle represent changes in either cell size or cell numbers, but for resource-based interactions, biomass should more directly reflect resource consumption and the impact on other species than cell numbers.

The number of doublings, that is, generations, was estimated by summing *log*_*2*_(OD.end/OD.start) for each growth period in each microcosm. Across species in monocultures, this ranged from 62 to 91 in beech tea, 60 to 87 in pH5 tea and 21 to 70 generations in spruce tea for each species. The mean number of generations after transfer to the new environments was 56.2 in beech and pH5 tea and 28.3 in spruce tea. Note that this calculation assumes cell sizes were constant during each growth period but not among species or over the whole duration of the experiment (that is, if the scaling constant to convert from OD to number of cells is *x* in one time period, it cancels in x*OD.end/x*OD.start).

### Growth assays to measure changes in species yields

We measured evolution of constituent species by isolating them from final microcosms and performing growth assays together with stored ancestral isolates (those used to initiate the experiment). Aliquots of all microcosms were plated onto R2A plates in order to detect the presence of each species. We estimate a lower detection limit of ∼1 in 10 000. Samples of all species present were isolated based on their colony morphology and the identity of species that co-occurred with others of similar morphology checked by sequencing 16S (of 85 such isolates, 71 were identified correctly by morphology, and the remainder were reassigned to the correct identification; Supplementary Table S2). Isolates were stored in glycerol at −80 °C. For growth assays, frozen samples of ancestral and evolved isolates were re-inoculated into 150 μl of the medium they grew on during the experiment. OD was measured every 24 h for 5 days. We thereby measured whether species evolved generally increased growth yields, and whether the extent of changes varied among microcosms.

### Statistical analyses

We used linear models to analyse productivity–richness relationships in each environment in the initial growth period following transfer into the separate environments. To infer species interactions, we compared observed community yields *A*_*j*_ to the sum of monoculture yields of constituent species, Σ*a*_*i*_*d*_*i,j*_ where *a*_*i*_ is the monoculture yield of species *i* and *d*_*i,j*_ indicates presence (1) or absence (0) of species *i* in community *j*. A value of the ratio *A*_*j*_/Σ*a*_*i*_*d*_*i,j*_ of 1 indicates that community yields are additive and hence there is no interaction, <1 indicates a negative interaction and >1 indicates a positive interaction (Figure 1a). To test for variation in species interactions among treatments, we fitted a linear model of community yields against the sum of monoculture yields, *A*_*j*_*=β*_*t=*0_(*E,S,*Σ*a*_*i*_*d*_*i,j*_), with slopes and intercepts that vary with environment, *E*, and log(richness), *S.* We simplified the model using stepwise removal (step function in R (R Core Team, 2014)). We call this baseline ‘model 0'. Under neutrality, we expect slope=1 and intercept=0; fitted values summarise the pattern of interactions in each treatment.

We used a linear mixed effects model (using lme in the nlme library in R (Pinheiro *et al.,* 2014)) to investigate whether the productivity–richness relationship in each environment changed over time. Fixed effects were time, species richness, environment and their two- and three-way interactions, and the random effect was microcosm that takes account of pseudoreplication due to repeated measures (Lindstrom and Bates, 1990). We fitted the model using maximum likelihood and simplified fixed effects using the stepAIC function in MASS. The simplified model was refitted using REML to report results. We used further models and data manipulation to explore the possible cause of changes.

(1) To estimate the changes caused by extinction (Figure 1b, case i), we used a modified version of model 0 to estimate predicted community yields at week 5 assuming that the only change was species extinction: *A*_*j,t=*5_*=β*_*t=*0_(*E,S,*Σ*a*_*i,t=*0_*d*_*i,j*_*ɛ*_*i,j*_), where *ɛ*_*i,j*_ is a binary term indicating survival (1) or extinction (0) of species *i* in community *j*. We called this model 1. By keeping slopes and intercepts the same as week 0, we assumed no net changes in species interactions over time. The accuracy of prediction was judged by regressing the observed change in community yields, *A*_*j,t=*5_−*A*_*j,t=*0,_ with the predicted change in community yields, *β*_*t=*0_(*E,S,*Σ*a*_*i*_*d*_*i,j*_*ɛ*_*i,j*_)−*A*_*j,t=*0_, and summarised by adjusted *R*^2^ and the Akaike information criterion (AIC).

(2) Similarly, to estimate changes caused by additive evolution of species yields, we constructed model 2 that predicted *A*_*j,t=*5_ as *β*_*t=*0_(*E,S,*Σ*a*_*i,t=*5_*d*_*i,j*_) assuming the only change was in monoculture yields. We repeated this using species yields measured from final isolates of species from each community at week 5, *β*_*t=*0_ (*E,S*,Σ*b*_*i,j,t=*5_ *d*_*i,j*_), called model 3. We selected the model yielding the lowest AIC for the regression between observed and predicted changes in community yields as the preferred model for additive evolution in species yields. We also constructed models with combinations of extinction and additive evolution in species yields, for example, *β*_*t=0*_(*E,S*,Σ*b*_*i,j,t=*5_ *d*_*i,j j*_*ɛ*_*i,j*_), and compared all models using AIC.

(3) Selecting the best model from those above, we fitted a new version of that model assuming that mean species interactions within environment and richness treatments also changed, that is, fitting new slopes and intercepts. As model 2 was best (see Results), we fitted *β*_*t=*5_(*E,S*,Σ*a*_*i,t=*5_ *d*_*i,j*_) that we call model 4. The additional variation explained by adding evolution of species interactions was calculated as the increase in adjusted *R*^2^.

The above approach allows separate effects to be teased apart but does so by splitting up the data. To test whether species interactions changed over time using all data, we fitted a linear mixed effects model to observed community yields at the start (*t*=0 at the start of the environmental treatments) and end (*t*=5 weeks): *A*_*j,t*_*=β*(*E,S*,*t*, Σ*a*_*i,t=*5_ *d*_*i,j*_) with microcosm as a random effect. We compared the model with one without time using analysis of variance. A significant effect of time indicates changes in interaction strengths: changes due to additive evolution of species yields would be accounted for by the final term Σ*a*_*i,t=5*_*d*_*i,j*_ instead.

## Results

### Productivity–richness relationship immediately after transfer to new environments

**Figure 2:**
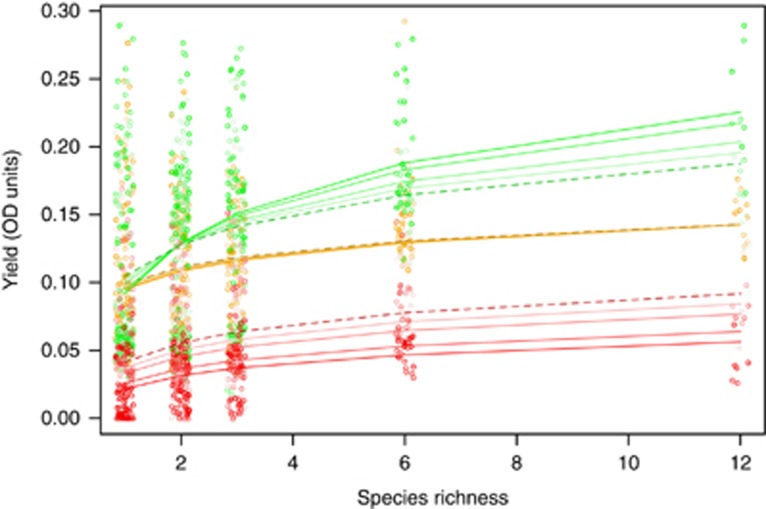
The relationship between yield following a serial transfer event and the number of species in a microcosm. Orange=beech tea medium; green=pH5 beech tea medium; red=spruce tea medium. The dashed lines indicate the fitted curve for yields immediately after being transferred into the different environments (that is, after 2 weeks of culturing on beech tea). Solid lines are the fitted curves for later time periods during the experiment: 1, 2, 4 and 5 weeks after transfer to the new environments respectively for increasingly darker lines. Curves become steeper over time in pH5 medium and lower over time in spruce tea.

In the first growth period following transfer into the different environments, microcosms displayed a classical increase in community yield with increasing richness, but the slope differed among environments (dashed lines, Figure 2, interaction between environment and log.richness, F_2, 327_=3.1, *P*=0.047; Supplementary Table S3). Yields on spruce tea (in red) were uniformly lower across all microcosms than in beech tea (in orange, *t*=−7.5, d.f.=327, *P*<0.0001). The slope of the curve was similar in beech and spruce tea (*t*=0.76, d.f.=327, *P*=0.4). In contrast, yields in the pH5 medium (in green) were similar to beech tea in monocultures (*t*=0.3, d.f.=327, *P*=0.8) but was higher and increased more steeply in diverse microcosms relative to control beech tea (slope of yield with richness in pH5, *t*=2.4, d.f.=327, *P*=0.016).

**Figure 3:**
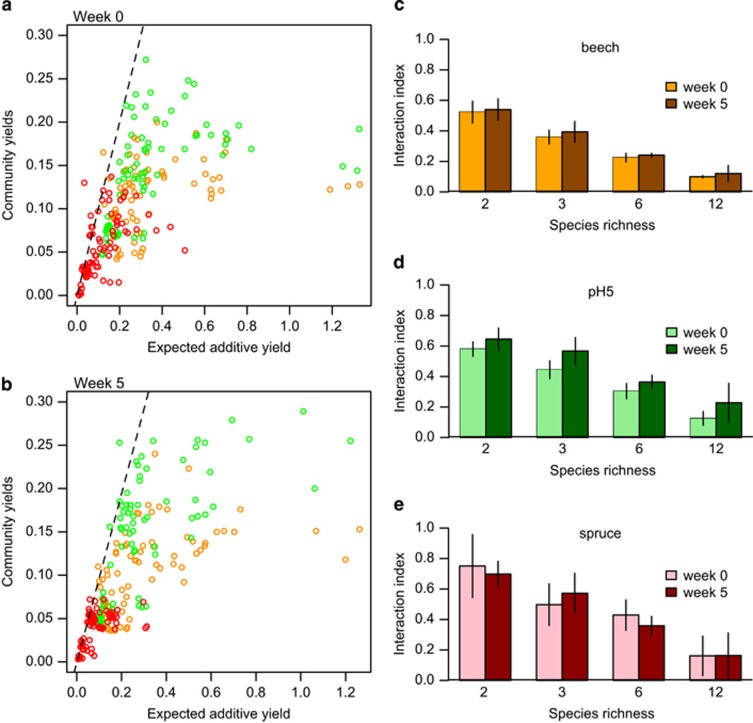
(**a**) The observed yields for communities with two or more species at the start plotted against the sum of the monoculture yields of constituent species. Orange=beech tea medium; green=pH5 medium; red=spruce tea medium. The dashed line shows the 1:1 relationship. (**b**) The observed yields for communities with two or more species at the end plotted against the sum of the yields of constituent species measured from community isolates. The proportion of communities displaying synergistic growth did not change over time (4.0% in week 0 versus 3.6% in week 5, *z*=−0.25, d.f.=448, *P*=0.81, general linear model with binomial errors) but was significantly higher in spruce tea than the other media (9.3% communities versus 1.3% *z*=2.7, d.f.=446, *P*=0.007) and in less diverse microcosms (*t*=−2.4, d.f.=446, *P*=0.019). (**c**) Mean interaction index, calculated as the ratio of observed to expected additive yields, shown across richness levels and for the start and end of the experiment in beech tea. A value of 1 would be additive growth, that is, no interaction, and values of <1 indicate negative interactions. The 95% confidence intervals are shown. (**d**) Mean interactions in pH5 tea. (**e**) Mean interactions in spruce tea.

The shape of increase in yields with species richness was consistent with generally negative species interactions. The yields of two-species mixtures, for example, were only on average 52.3±7.1%, 58.0±4.8% and 75.1±20.7% of the expected additive yields in the control beech, pH5 and spruce media, respectively (*t*-tests of the ratio of observed and additive yields repeated for dicultures in each environment in turn, *t*=−13.5, −17.8 and −2.5, d.f.=35, *P*<0.0001, *P*<0.0001 and *P*=0.019, respectively, Figure 3a). Yields were even lower relative to additive expectation in more diverse microcosms (for example, ranging between 9.9% and 16.1% in the 12-species communities; in a linear model, the slope with additive prediction becomes shallower at higher richness; *t*=−9.6, d.f.=219, *P*<0.0001, Supplementary Table S4). An additional 25.5% of variation was explained by composition and its interaction with environment, indicating that the community yield also depended on which combinations of species were cultured together (composition, F_24, 149_=25.7, *P*<0.0001; interaction with environment, F_48, 149_=6.1, *P*<0.0001).

### Changes in the productivity–richness relationship over time

The relationship between community yields and richness changed relatively little over time in control beech tea (Figure 2, increasingly darker lines indicate fitted curves for later time points, Supplementary Table S5). There was no significant change in average yields at any level of richness in the control beech tea. There was a significant decline in average yields in spruce tea (linear mixed effects model, *t*=−2.4, d.f.=1320, *P*=0.017), equating to a 30% decline across richness levels. In contrast, the relationship in the pH5 medium became significantly steeper during the course of the experiment (interaction between time and richness in the pH5 treatment, coefficient=2.43 × 10^−5^, *t*=2.97, d.f.=1320, *P*=0.003). The more diverse communities now grew better than had the ancestral communities, whereas there was no significant change in average yields of monocultures over time (effect of time in pH5 environment, just monocultures included, *t*=−1.1, d.f.=141, *P*=0.26). We explore three possible causes for the change in relationships through time in the subsequent sections.

### Ecological sorting

The first possible cause of changes in community growth over time is that ecological sorting led to the loss of species in some communities (Figure 1a, case i). Across all environments, species extinction rates increased from nearly 0 in monocultures to 74% in 12-species communities on control beech tea (Supplementary Figure S1). Extinction rates were significantly higher in pH5 and spruce tea media than in control beech tea (84% and 86%, respectively, in 12-species communities, *z*>3, d.f.=326, *P*<0.005 for both comparisons, generalised linear model with binomial errors). Extinction alone, however, was insufficient to explain changes in community yields: predicted changes in yields due to loss of species alone explained 0.35% of the variation in the changes in community yields between the start and the end of the experiment (a nonsignificant fraction; model 1, Table 2 and Supplementary Figure S2a).

### Evolution of species yields

The second possible cause of changes in community growth over time is that yields of constituent species changed but the strength of interactions between them remained constant (Figure 1b, case ii: additive evolution). For example, if the species with highest yields evolved even higher yields, this could lead to a disproportionate increase in yield of more diverse communities (which are more likely to include them). Some monocultures did evolve higher yields on average by the end of the experiment (for example, species THB22 and 39 in beech tea; Supplementary Figure S3). In spruce tea, monocultures had lower yields at the end than at the start (mixed effects model, effect of time in spruce treatment, *t*=−2.5, d.f.=426, *P*=0.014; Supplementary Figure S3). The change in community yields during the experiment was significantly predicted by the change in monoculture yields (explaining 17.7% of the variation; model 2, Table 2 and Supplementary Figure S2).

**Figure 4:**
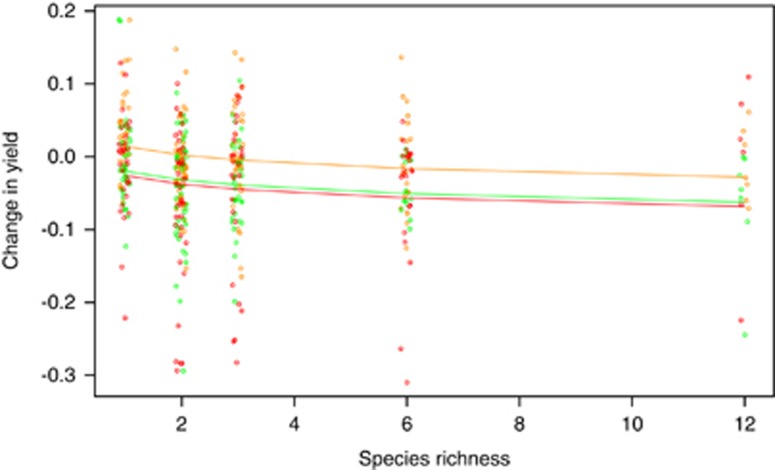
The observed (dots) and fitted (lines) yields of species isolates from communities relative to the growth rate of ancestral isolates. Orange=beech tea; green=pH5 beech tea; red=spruce tea. Species that evolved in more diverse communities have significantly lower yields (and less on average than the ancestral isolates) when subsequently grown in monoculture than those from less diverse communities.

A further complication is that species might have evolved different yields in communities than in monocultures, so that monocultures at the end might not reflect changes in yields of component species in communities. We isolated all the surviving species from each microcosm at the end, regrew them in monoculture and then tested whether their yields in isolation varied significantly depending on the species richness of the community they had evolved in. On average, yields of isolates declined with increasing diversity of the source community in all three environments (*t*=−3.2, d.f.=421, *P*=0.0015, Figure 4). The change in yield relative to the ancestral isolates was lower in pH5 tea and spruce tea than in control beech tea across all diversity levels (*t*=−4.0 and −4.6, respectively, d.f.=421, *P*<0.0001). Replacing yields for surviving species with those measured from final community isolates did not improve the predictability of community yields (model 3, Table 2 and Supplementary Fig. S2; explains 17.0% of variation, AIC=−903.1; model 2 using yields measured from monoculture isolates, AIC=−905.2).

**Figure 5:**
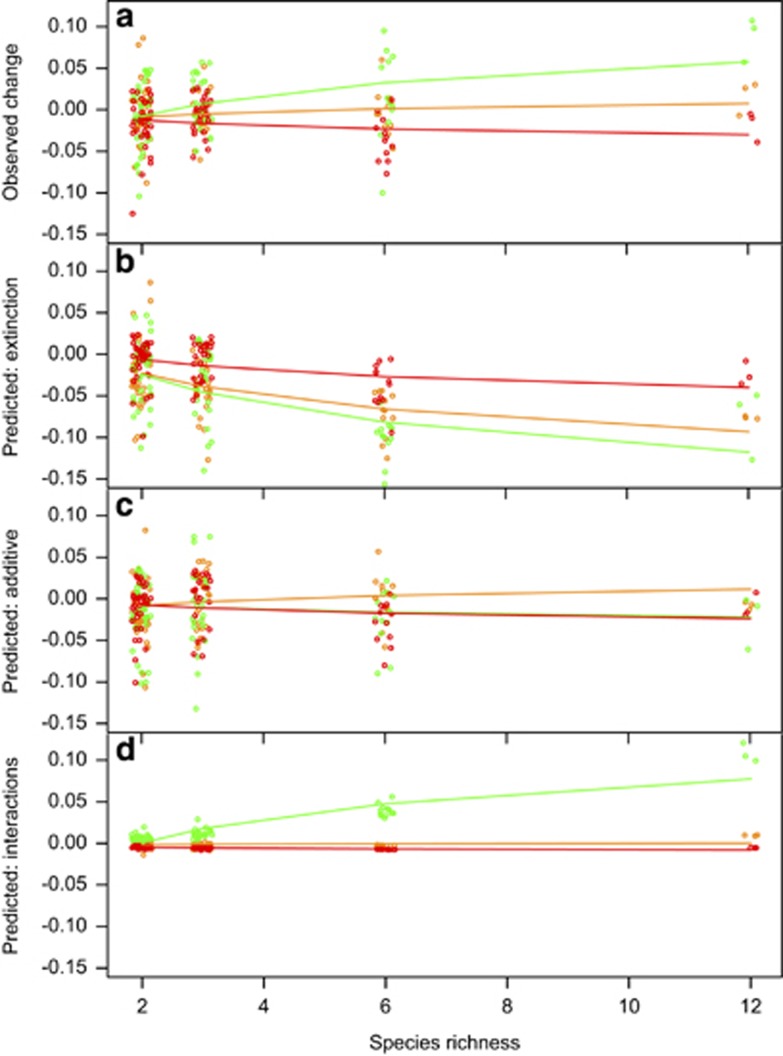
(**a**) Observed changes in community yields, *A*_*i,t=*5_−*A*_*i,t=*0_, between the start and the end of the experiment extracted from data in Figure 2. Predicted changes in community yields because of (**b**) species extinction from model 1 in Table 2, (**c**) additive evolution of species yields from model 2 and (**d**) systematic changes in species interactions within environment and richness treatments, calculated as predicted changes from model 4 minus predicted changes from model 2. Additive evolution reproduces the changes in control beech tea and spruce tea, but evolution of species interactions is needed to reproduce the changes in pH5 tea.

Although additive evolution of species yields explained changes in the productivity–richness relationship in beech tea and spruce tea over time, it could not alone explain the steepening productivity–richness relationship in pH5 tea (Figure 5c).

### Evolution of species interactions

The final explanation for observed changes in community yields over time is that species interactions evolved during the experiment (Figure 1b, case ii). We plotted the yields of communities at the end against the sum of monoculture yields at the end (Figure 3b) and compared patterns with those at the start of the experiment. There was no consistent change in the strength of interactions in control beech tea and spruce tea (Figures 3c and e): observed changes of community yields in spruce tea were predicted well by the sum of species yields. In contrast, interactions became less negative in pH5 tea: observed yields were higher relative to additive yields than they had been at the start, across all richness levels (Figure 3d). In a linear mixed effects model, the slope between observed and additive yields in pH5 tea became less negative over time (*t*=2.86, d.f.=215, *P*=0.0046, Supplementary Table S6). Allowing model coefficients to vary with time, that is, for species interactions to change systematically within environment and richness treatments, explained nearly twice as much variation (model 4, Table 2, explained 33.9%) as the best model with fixed species interactions (model 2), and was necessary to explain the observed steepening of the productivity–richness relationship in pH5 tea (Figure 5d).

To conclude, 0.35% of variation in the change in community yields between the start and the end of the experiment was explained by species extinction, 17.7% by additive evolution of species yields and 14.3% (=32.0–17.7%) by systematic changes in species interactions within richness and environment treatments. The latter fraction was responsible for the steepening of the productivity–richness relationships over time in pH5 tea. A further 47.5% of variation in the change in community yields was explained by changes in species interactions within particular compositions and environments (model 5, Table 2). The remaining 20.5% was residual variation among the 3 replicates of each composition by environment treatment.

### Sensitivity analyses

We performed two further analyses to check sensitivity to our assumptions. First, assuming that species that had gone extinct by the end were already contributing little to community yields at the start, we repeated the above analyses but excluded species that were extinct by the end from the calculation of sum of monoculture yields at the start and used final richness instead of starting richness. Conclusions were the same as reported above (Supporting Results S1, Supplementary Table S7). Second, to check whether results were because of the dominant effects of a few species, we fitted alternative models with separate coefficients for each species, that is, *A*_*j*_=*β*(*E,S,t,a*_*i*_*d*_*i,j*_). A simplified linear mixed effect model of community yields over time retained terms for yields of all the species in the model (Supplementary Table S8). Focusing on pH5 tea, coefficients increased in THB6, 20, 22 and 39 over time, whereas no species displayed decreased coefficients in the simplified model (Supplementary Table S9), and hence the less negative interactions over time.

## Discussion

Our results show that diversity affected how both collective growth of communities and yields of constituent species changed over time. The largest effect occurred in pH5 media, in which the relationship between community yields and diversity, already steeper than in the other media at the start, became even steeper by the end. One possible explanation for this change is that one or a few species evolved higher yields over time, and because they are more likely to be contained in more diverse microcosms than in less diverse microcosms, this would steepen the relationship. Partitioning the change into separate effects, we found that changes in beech tea and spruce tea could indeed be explained by adding together the effects of observed changes of each species in monoculture. However, this was insufficient to explain the increase in community yields in pH5 tea. Instead, interactions between species must have changed: communities now grew better relative to the yields of their constituent species than expected based on the levels of interactions fitted at the start of the experiment. Although it has been found that coevolution might occur less in more diverse communities than in pairwise systems (Futuyma and Agrawal, 2009; terHorst, 2010), we found greater change in species interactions in more diverse microcosms.

Further information came from comparing patterns of community growth with the growth of constituent species. When species were isolated from communities at the end of the experiment, their yields were lower when isolated from more diverse communities. One explanation for this finding is that competition reduced the rate of adaptation to new conditions in those media and led to lower final yields, as predicted in the models of de Mazancourt *et al.* (2008) and Johansson (2008). By this mechanism alone (that is, adding up separate species effects), the growth rate of communities containing those species should be lower than the growth rate of the best growing species in monoculture. Instead, in pH5 tea, community yields evolved to be higher in more diverse microcosms and more so than predicted by the effects of adding together changes in yields of the surviving constituent species. This is consistent with the alternative that species evolved less negative interactions. For example, the decline in yields of constituent species when grown in isolation might reflect increased specialisation on a subset of resources relative to those used by monoculture isolates of the same species. Although testing this mechanism was impractical for the number of microcosms here, we demonstrated it previously using spectroscopy and competition assays in a community of five of the same species studied here (Lawrence *et al.,* 2012).

Another possible mechanism, again demonstrated in the previous study of Lawrence *et al.* (2012), is that species evolved to use the waste products of other species. This could lead to higher collective growth than expected from separate growth of isolates in monocultures. Without direct evidence of resource use here, however, there could be other mechanisms such as synergistic benefits of excreted enzymes for breaking down substrates or benefits of metabolising or otherwise neutralising toxic compounds in the medium, such as tannins. We had no evidence that phage were present in our cultures, but in principle apparent competition mediated by phage could also have played a role (Brockhurst *et al.,* 2006). Separating the role of alternative interactions in determining overall patterns of community yields would be an interesting topic for future study.

Further work would be needed to identify specific mechanisms for the patterns we observed. We initially chose environmental treatments to differ in two different ways: a change in physical environment (pH) and in resource availability (the greater carbon/nitrogen ratio and different secondary compounds of spruce needles compared with beech leaves). As expected, both species and communities grew far slower initially on spruce tea than on control beech tea and indeed never evolved to cope well with this medium: this could reflect having half as many generations in spruce tea and hence less chance to adapt than in the other environments. In contrast, pH5 tea displayed equivalent yields of monocultures but higher yields of diverse microcosms initially. In general terms, pH5 might have modified chemistry of the tea and freed up additional resources that species used differently to one another. Without detailed chemical investigation, which was prohibitive for the number of species and microcosms here, we cannot explain why patterns differed between environments. Future work on how microbial communities respond to new environments will require greater understanding of specific mechanisms, including quantification of resource use and other interactions (c.f., Turnbull *et al.,* 2013), for example by using artificial media containing known resources (Replansky and Bell, 2009; Langenheder *et al.,* 2010) that would facilitate assays of resource use. Tracking species abundances in detail over time would also be useful in future to reconstruct changes in ecological interactions.

To conclude, diversity not only improved productivity as measured by total community biomass yield at the start of the experiment, but also promoted the evolution of improved productivity in the pH5 media over time. The timescales of ∼60 generations are relevant for short-term changes in microbial communities and similar processes could operate over decades in annual organisms responding to current climate change. Our microcosms contained less diversity than real communities and no immigration, and hence there was less scope for ecological sorting than would be expected in natural tree-holes. Real communities are open to colonisation and hence diversity might be preserved by immigration of preadapted types previously found in other areas, for example those with already lower optimum pH. There was also potentially more scope for evolution because of serial transfer. In wild tree-holes, resources will arrive more sporadically and species will alternate between high-resource and low-resource conditions on a haphazard basis. We now need studies of wild or semi-wild communities to understand how function changes in new environments.

## Figures and Tables

**Table 1:**
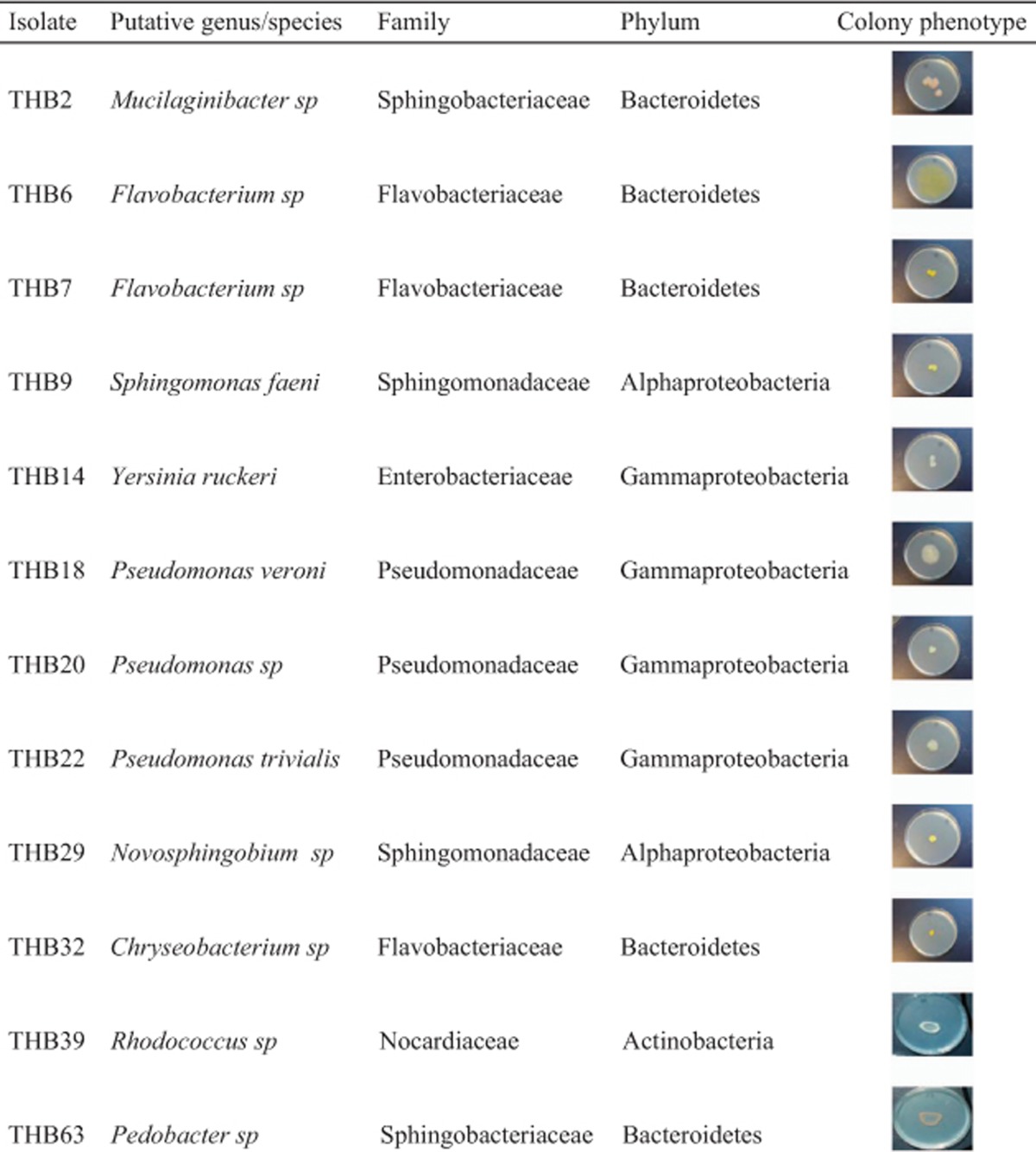
Species isolates used in the experiments

**Table 2:** Comparison of alternative predictors of the change in community yields between week 0 and week 5

	*Predictor of final yields*	*Description*	*AIC*	*Adjusted* R^*2*^	t	P
1	*β*_*t=*0_ (*E,S*, Σ*a*_*i,t=*0_ *ɛ*_*i,j*_)	Extinction only	−862.0	0.0035	1.33	0.18
2	*β*_*t=*0_ (*E,S*, Σ(*a*_*i,t=*5_ *d*_*i,j*_)	Monoculture changes only	−905.2	0.177	7.02	<0.0001
3	*β*_*t=*0_ (*E,S*, Σ(*b*_*i,j,t=*5_ *d*_*i,j*_)	Monoculture changes only (measured from community isolates)	−903.1	0.170	6.84	<0.0001
4	*β*_*t=*5_ (*E,S*, Σ(*a*_*i,t=*5_ *d*_*i,j*_)	Monoculture plus species interactions changes	−948.0	0.320	10.3	<0.0001
5	*β*_*t=*5_ (*E,C*, Σ(*a*_*i,t=*5_ *d*_*i,j*_)	Monoculture+species interactions by composition	−1218	0.795	29.5	<0.0001

Abbreviation: AIC, Akaike information criterion.

*a*_*i,t=x*_: monoculture yields of species *i* at week *x*; *β*_*t=x*_(*E,S*): slopes fitted between community yields and Σ(*a*_*i,t=*0_ *d*_*i,j*_) at week *x* in each environment, *E*, and richness level, *S*; *d*_*i,j*_: the presence (1) or absence (0) of each species in community *j* at the start; *ɛ*_*i,j*_: the survival (1) or extinction (0) of each species in community *j* by the end; *b*_*i,t=*5_: monoculture yields of isolates of species *i* extracted from community *j* at week 5; *C*: a factor with levels denoting each community composition.

## References

[bib1] Awasthi A Singh M Soni SK Singh R Kalra A 2014 Biodiversity acts as insurance of productivity of bacterial communities under abiotic perturbations ISME Je-pub ahead of print 13 June 2014; doi:10.1038/ismej.2014.91 PMC4260711 24926862

[bib2] Bell G 2007 The evolution of trophic structure Heredity 99 494 505 17687253 10.1038/sj.hdy.6801032

[bib3] Bell T Lilley AK Hector A Schmid B King L Newman JA 2009 A linear model method for biodiversity-ecosystem functioning experiments Am Nat 174 836 849 19842969 10.1086/647931

[bib4] Bell T Newman JA Silverman BW Turner SL Lilley AK 2005 The contribution of species richness and composition to bacterial services Nature 436 1157 1160 16121181 10.1038/nature03891

[bib5] Brockhurst MA Fenton A Roulston B Rainey PB 2006 The impact of phages on interspecific competition in experimental populations of bacteria BMC Ecology 6 19 17166259 10.1186/1472-6785-6-19 PMC1764007

[bib6] Connolly J Bell T Bolger T Brophy C Carnus T Finn JA 2013 An improved model to predict the effects of changing biodiversity levels on ecosystem function J Ecol 101 344 355

[bib7] de Mazancourt C Johnson E Barraclough TG 2008 Biodiversity inhibits species' evolutionary responses to changing environments Ecol Lett 11 380 388 18248449 10.1111/j.1461-0248.2008.01152.x

[bib8] Foster KR Bell T 2012 Competition, not cooperation, dominates interactions among culturable microbial species Curr Biol 22 1845 1850 22959348 10.1016/j.cub.2012.08.005

[bib9] Futuyma DJ Agrawal AA 2009 Macroevolution and the biological diversity of plants and herbivores Proc Natl Acad Sci USA 106 18054 18061 19815508 10.1073/pnas.0904106106 PMC2775342

[bib10] Gravel D Bell T Barbera C Bouvier T Pommier T Venail P 2011 Experimental niche evolution alters the strength of the diversity-productivity relationship Nature 469 89 92 21131946 10.1038/nature09592

[bib11] Griffiths RI Thomson BC James P Bell T Bailey M Whiteley AS 2011 The bacterial biogeography of British soils Environ Microbiol 13 1642 1654 21507180 10.1111/j.1462-2920.2011.02480.x

[bib12] Hooper DU Chapin FS Ewel JJ Hector A Inchausti P Lavorel S 2005 Effects of biodiversity on ecosystem functioning: a consensus of current knowledge Ecol Monogr 75 3 35

[bib13] Isbell F Calcagno V Hector A Connolly J Harpole WS Reich PB 2011 High plant diversity is needed to maintain ecosystem services Nature 477 199 U196 21832994 10.1038/nature10282

[bib14] Johansson J 2008 Evolutionary responses to environmental changes: how does competition affect adaptation Evolution 62 421 435 18031306 10.1111/j.1558-5646.2007.00301.x

[bib15] Kalbitz K Kaiser K Bargholz J Dardenne P 2006 Lignin degradation controls the production of dissolved organic matter in decomposing foliar litter Eur J Soil Sci 57 504 516

[bib16] Langenheder S Bulling MT Solan M Prosser JI 2010 Bacterial biodiversity-ecosystem functioning relations are modified by environmental complexity PLoS One 5 10.1371/journal.pone.0010834 PMC2877076 20520808

[bib17] Lawrence D Fiegna F Behrends V Bundy JG Phillimore AB Bell T 2012 Species interactions alter evolutionary responses to a novel environment PLoS Biol 10 e1001330 22615541 10.1371/journal.pbio.1001330 PMC3352820

[bib18] Lindstrom MJ Bates DM 1990 Nonlinear mixed effects models for repeated measures data Biometrics 46 673 687 2242409

[bib19] Liow LH Van Valen L Stenseth NC 2011 Red Queen: from populations to taxa and communities Trends Ecol Evol 26 349 358 21511358 10.1016/j.tree.2011.03.016

[bib20] Loeuille N 2010 Influence of evolution on the stability of ecological communities Ecol Lett 13 1536 1545 21054734 10.1111/j.1461-0248.2010.01545.x

[bib21] Loreau M Hector A 2001 Partitioning selection and complementarity in biodiversity experiments Nature 412 72 76 11452308 10.1038/35083573

[bib22] Mori AS Furukawa T Sasaki T 2013 Response diversity determines the resilience of ecosystems to environmental change Biol Rev Camb Philos Soc 88 349 364 23217173 10.1111/brv.12004

[bib23] Myers JA Curtis BS Curtis WR 2013 Improving accuracy of cell and chromophore concentration measurements using optical density BMC Biophysics 6 4 24499615 10.1186/2046-1682-6-4 PMC3663833

[bib24] Norberg J Swaney DP Dushoff J Lin J Casagrandi R Levin SA 2001 Phenotypic diversity and ecosystem functioning in changing environments: a theoretical framework Proc Natl Acad Sci USA 98 11376 11381 11535803 10.1073/pnas.171315998 PMC58737

[bib25] Osmond MM de Mazancourt C 2013 How competition affects evolutionary rescue Phil Trans R Soc Lond B 368 20120085 20120085 23209167 10.1098/rstb.2012.0085 PMC3538452

[bib26] Pekkonen M Ketola T Laakso JT 2013 Resource availability and competition shape the evolution of survival and growth ability in a bacterial community PLoS One 8 e76471 24098791 10.1371/journal.pone.0076471 PMC3787024

[bib27] Pinheiro J Bates D DebRoy S Sarkar D R Core Team 2014 nlme: Linear and nonlinear mixed effects models. R package version 3.1-118http://CRAN.R-project.org/package=nlme.

[bib28] Price TD Kirkpatrick M 2009 Evolutionarily stable range limits set by interspecific competition Proc R Soc Lond B 276 1429 1434 10.1098/rspb.2008.1199 PMC2677220 19324813

[bib29] R Core Team 2014 R: A Language and Environment for Statistical Computing R Foundation for Statistical Computing: Vienna, Austria , http://www.R-project.org/ .

[bib30] Reasoner DJ Blannon JC Geldreich EE 1979 Rapid 7-Hour fecal coliform test Appl Environ Microbiol 38 229 236 42349 10.1128/aem.38.2.229-236.1979 PMC243471

[bib31] Reich PB Tilman D Isbell F Mueller K Hobbie SE Flynn DFB 2012 Impacts of biodiversity loss escalate through time as redundancy fades Science 336 589 592 22556253 10.1126/science.1217909

[bib32] Replansky T Bell G 2009 The relationship between environmental complexity, species diversity and productivity in a natural reconstructed yeast community Oikos 118 233 239

[bib33] Steudel B Hector A Friedl T Loefke C Lorenz M Wesche M 2012 Biodiversity effects on ecosystem functioning change along environmental stress gradients Ecol Lett 15 1397 1405 22943183 10.1111/j.1461-0248.2012.01863.x

[bib34] terHorst CP 2010 Evolution in response to direct and indirect ecological effects in pitcher plant inquiline communities Am Nat 176 675 685 20955011 10.1086/657047

[bib35] Turnbull LA Levine JM Loreau M Hector A 2013 Coexistence, niches and biodiversity effects on ecosystem functioning Ecol Lett 16 116 127 23279851 10.1111/ele.12056

