# Supplementary material for: Evolution of species interactions determines microbial community productivity in new environments
Source: ISME J. 2014 Nov 11;9(5):1235–45. doi: 10.1038/ismej.2014.215 (PMC4409166; doi:10.1038/ismej.2014.215)
Supplement: Supplementary Information [file ismej2014215x1.pdf]

SUPPLEMENTARY FIGURES

FIGURE S1

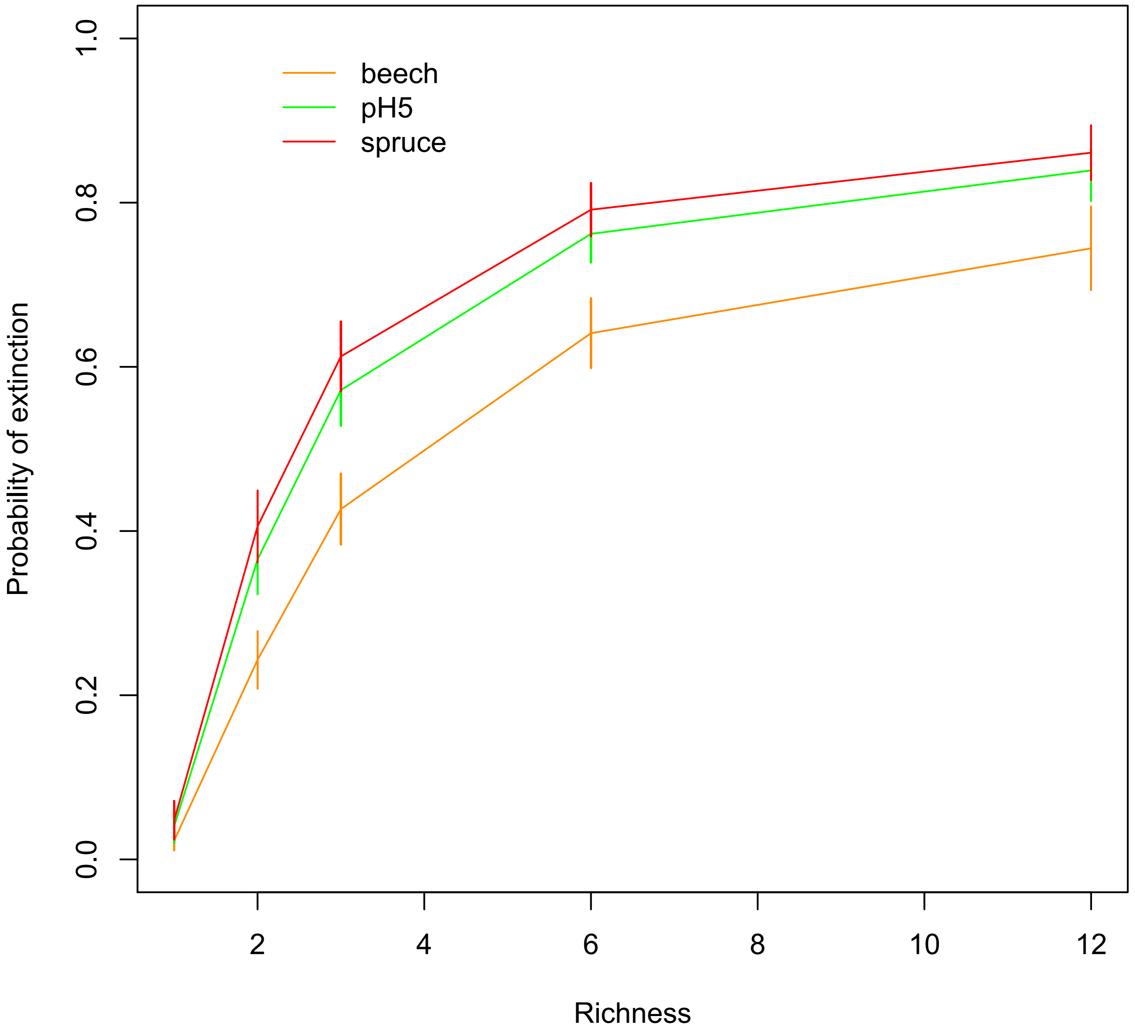

Figure S1. The probability of extinction per species during the experiment in communities of different species richness and in the three environments: orange = beech tea; green = pH5 beech tea and red = spruce tea. Predicted probabilities with standard errors from a general linear regression with binomial errors are shown, treating richness as a factor.

FIGURE S2

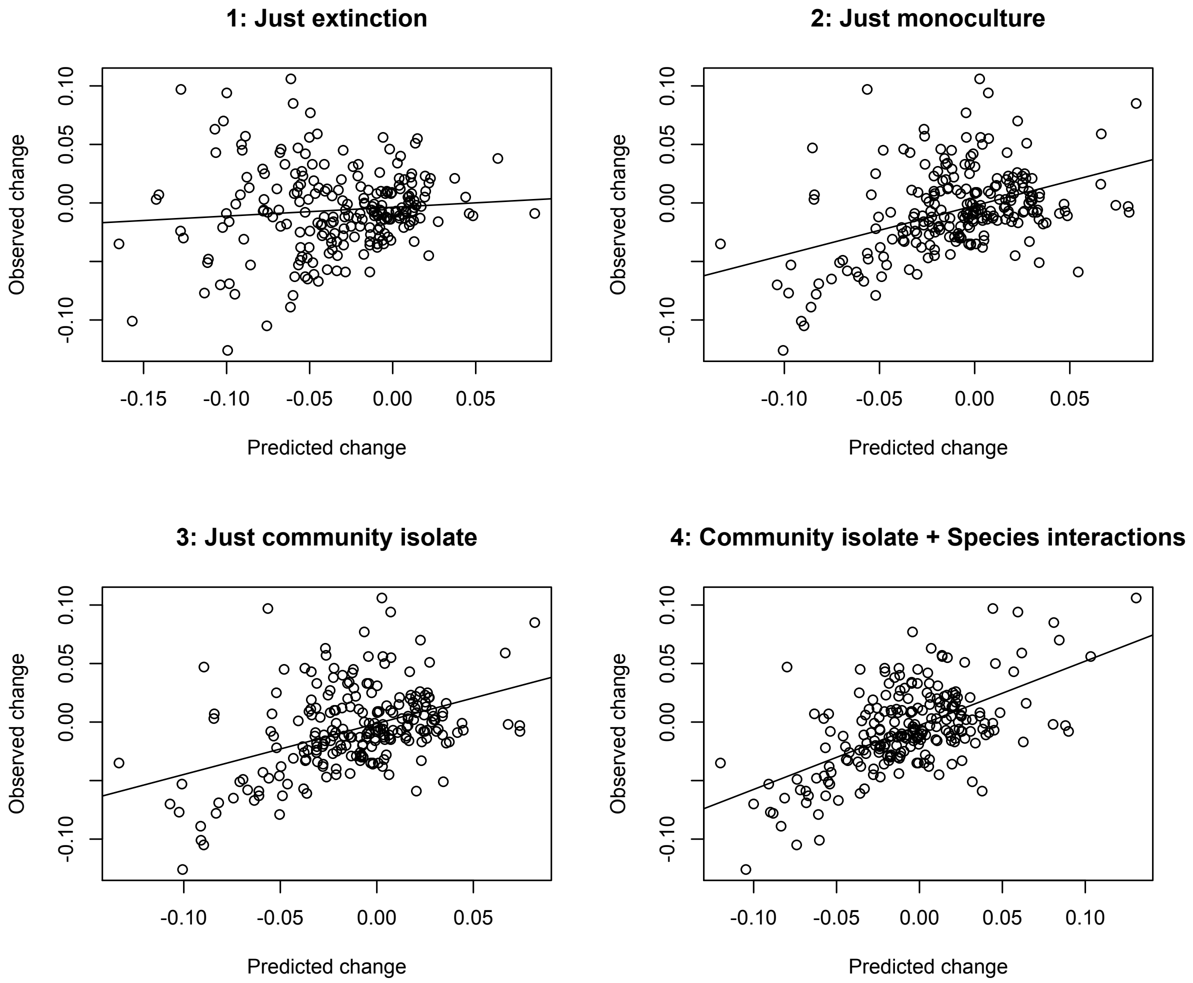

Figure S2. The observed change in community yields plotted against the four alternative predictors of community changes outlined in table 2. Model 1 predicts changes if the only change between week 0 and week 5 was the loss of species that were extinct by the end. Model 2 predicts changes based solely on changes in monoculture yields between week 0 and week 5. Model 3 uses species isolates from final communities to estimate changes in the sum of monoculture yields for each community. Model 4 uses the same predictor as model 2, but fits a new slope for each environment X richness combination, i.e. that the strength of species interactions has changed. Full descriptions are given in table 2 and main text.

FIGURE S3

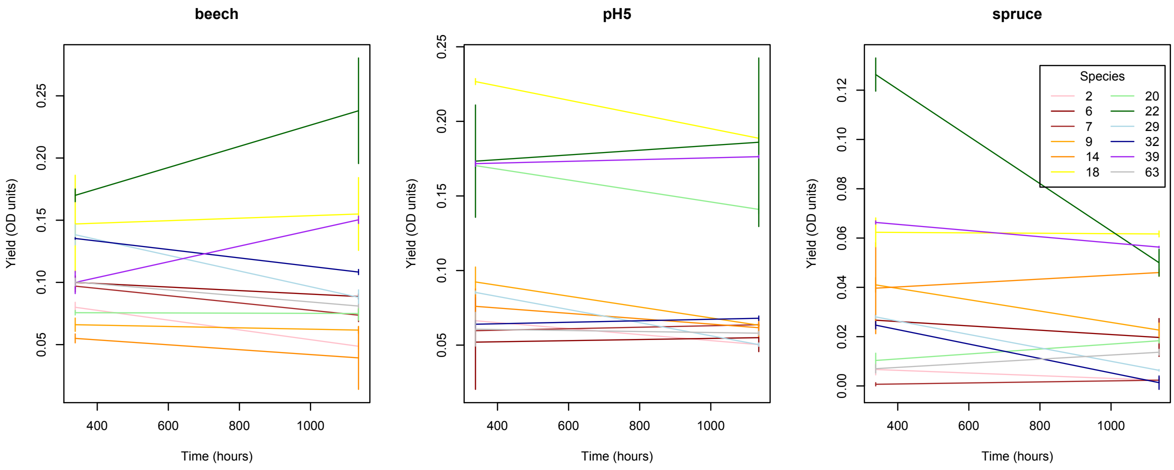

Figure S3. The yields of each species in monoculture at the start and end of the experiment in the three environments: control beech tea, pH5 beech tea and spruce tea. Standard errors are shown.

Supplementary tables

Table S1 - Composition of the experimental communities. Species numbers refer to numbers in table 1, e.g. 2,6 indicates that THB2 and THB6 were present. Composition Y had all 12 species together. Monocultures were labelled by their species number.

Composition	Species	Composition	Species
A	2,6	M	2,22,29
B	22,39	N	9,18,39
C	29,63	O	7,20,63
D	9,18	P	6,14,32
E	14,32	Q	22,32,63
F	7,20	R	2,7,18
G	7,18	S	14,20,29
H	14,20	T	6,9,39
I	2,29	U	9,14,18,20,29,32
J	9,63	V	2,6,7,22,39,63
K	6,22	W	6,7,9,18,32,63
L	32,39	X	2,14,20,22,29,39

Table S2 - Results of 16S sequencing to check morphological identification of final isolates from high diversity treatments. Red = mis- or un-identified based on morphology. Final isolate name: Environment_CompositionReplicate_Morphological ID.

Starting isolate	Name	Final isolate	Starting isolate	Name	Final isolate
THB6	Flavobacterium	BT_V1_6	THB18 continued		ph5_W3_18
		BT_V3_6			pH5_Y1_18
		ST_V1_6			pH5_Y3_18
		ST_V2_6			ST_U1_18
		ST_V3_6			ST_U2_18
		ST_W1_6			ST_U3_18
		ST_W2_6			ST_W3_18

		ST_T1_9			ST_Y1_18
		ST_T2_9			ST_Y2_18
		ST_T3_9			ST_Y3_18
THB7	Flavobacterium	BT_W1_7			BT_Y2_14
		BT_W2_7			BT_Y3_14
		BT_W3_7			pH5_Y3_14
		BT_Y2_yell	THB22	Pseudomonas trivialis+B10	pH5_Y2_14
		BT_Y1_7_			ST_Y2_14
		ph5_Y3_7			pH5_Y2_18
		BT_Y1_6_			ph5_Y3_20
THB14	Yersinia ruckeri	BT_U1_14			ST_Y2_20
		BT_U3_14			BT_Y3_22
		BT_Y1_14			ph5_V1_22
		ph5_Y1_14			ph5_V2_22
		ph5_U1_14			ph5_V3_22
		ST_U1_14			ph5_X1_22
		ST_U2_14			ph5_X2_22
		ST_U3_14			ph5_X3_22
		ST_X1_14			pH5_Y1_22
		ST_X2_14			pH5_Y2_22
		ST_X3_14			ST_V2_22
		ST_Y1_14			ST_V3_22
		ST_Y3_14			ST_X2_22
THB18	Pseudomonas veroni	D1_18			ST_X1_22
		D3_18			ST_X3_22
		D1_181			ST_Y2_22
		D3_181	THB29	Novosphingobium	C3_29
		BT_U1_18			C3_291
		BT_U3_18			I1_29

BT_Y1_18			I2_29
BT_Y2_18			I3_29
BT_Y3_18			I1_291
ph5_U1_18			I2_291
ph5_U3_18			I3_291
ph5_W1_18	THB32	Chryseobacterium	BT_V1_7_
ph5_W2_18			

Table S3 – Linear model of microcosm yields at the start of the experiment following transfer into the three environments: $F_{5,327}$=52.0, $R2$=0.43, p<0.0001.

	Estimate	Standard Error	t	p
Intercept	0.1019	0.0063	16.22	<0.0001
log(richness)	0.0142	0.0065	2.19	0.0295
Treatment.pH5	0.0046	0.0089	0.52	0.6031
Treatment.spruce	-0.0667	0.0089	-7.51	<0.0001
log(richness):treatment.pH5	0.0224	0.0092	2.43	0.0157
log(richness):treatment.spruce	0.0070	0.0092	0.76	0.4486

Table S4. Linear model of microcosm yields based on the sum of species yields, richness and treatments at the start of the experiment

	Estimate	Standard Error	t	p
Intercept	0.0029	0.0111	0.27	0.791
Sum.species.yields	0.5541	0.0521	10.65	<0.0001
log(richness)	0.0094	0.0093	1.01	0.312
Treatment.pH5	0.0267	0.0057	4.73	<0.0001
Treatment.spruce	0.0086	0.0088	0.98	0.33
Sum.species.yields X richness	-0.1921	0.0199	-9.64	<0.0001

Table S5 – Linear mixed effects model of microcosm yields over the course of the experiment.

Fixed effects	Value	Standard Error	DF	t-value	p-value
Intercept	0.101281	0.007110	1321	14.24	<0.0001
Time	-0.000006	0.000006	1321	-1.05	0.2938
log(richness)	0.016784	0.007369	327	2.28	0.0234
Treatment.pH5	0.009676	0.010060	327	0.96	0.3369
Treatment.spruce	-0.051495	0.010054	327	-5.12	<0.0001
Time:log(richness)	0.000002	0.000006	1321	0.35	0.7228
Time:treatment.pH5	-0.000012	0.000008	1321	-1.47	0.1422
Time:treatment.spruce	-0.000019	0.000008	1321	-2.38	0.0174
log(richness):treatment.pH5	0.007254	0.010424	327	0.70	0.487
log(richness):treatment.spruce	0.006171	0.010420	327	0.59	0.5541
Time:log(richness):treatment.pH5	0.000024	0.000008	1321	2.97	0.003
Time:log(richness):treatment.spruce	-0.000010	0.000008	1321	-1.22	0.2219

Random effects	Standard Deviation
Intercept	3.90E-02
Residual	2.53E-02

Table S6 – Linear mixed effects model of microcosm yields over the course of the experiment against the sum of species yields. Species yields in week 5 were measured using monoculture isolates.

Fixed effects	Value	Standard Error	DF	t-value	p-value
Intercept	0.03392	0.01309	221	2.59	0.0102
Sum.species.yields	0.38376	0.04381	215	8.76	0
log(richness)	0.01613	0.00924	221	1.75	0.0822

treatment.pH5	0.01764	0.01251	221	1.41	0.1599
treatment.spruce	-0.02125	0.01296	221	-1.64	0.1024
time	-0.00001	0.00001	215	-1.48	0.1413
Sum.species.yields:log(richness)	-0.14424	0.01661	215	-8.68	0
Sum.species.yields:treatment.pH5	0.00439	0.02850	215	0.15	0.8776
Sum.species.yields:treatment.spruce	0.07222	0.05556	215	1.30	0.195
Sum.species.yields:time	0.00003	0.00002	215	1.25	0.2113
treatmentpH5:time	-0.00001	0.00001	215	-0.43	0.6673
treatmentspruce:time	0.00001	0.00001	215	0.75	0.4518
Sum.species.yields:treatment.pH5: time	0.00008	0.00003	215	2.86	0.0046
Sum.species.yields:treatment.spruce: time	-0.00010	0.00008	215	-1.33	0.1855

Random effects	Standard Deviation
Intercept	0.0258
Residual	0.0238

Table S7. Comparison of alternative predictors of the change in community yields between week 0 and week 5: alternative version to table 2. The baseline linear model at time 0 modeled community yields as $\beta_{t=0}\,(E,S,\,\Sigma a_{i,t=0}\,\varepsilon_{i,j})$, i.e. excluding monoculture yields of species that went extinct by the end. Final richness (i.e. $\Sigma\,\varepsilon_{i,j}$ for each community *j*) was used in all the models instead of starting richness. To test whether starting richness had an additional effect we fitted versions of model 8 including both starting and final richness: both variables were retained in the simplified model, which was preferred over a model with just final richness alone (ANOVA, F_6=7.2, p<0.0001).

	Predictor of final yields	Description	AIC	Adjusted R2	t	p
6	$\beta_{t=0}\,(E,S,\Sigma a_{i,t=5}\,\varepsilon_{i,j})$	Monoculture changes only	-915.6	0.215	7.88	<0.0001

7	$\beta_{t=0}\,(E,S,\Sigma b_{i,j,t=5}\ \varepsilon_{i,j})$	Monoculture changes only (measured from community isolates)	-911.8	0.201	7.58	<0.0001
8	$\beta_{t=5}\,(E,S,\ \Sigma a_{i,t=5}\ \varepsilon_{i,j})$	Monoculture plus species interactions changes	-952.3	0.333	10.9	<0.0001
9	$\beta_{t=5}\,(E,C,\ \Sigma a_{i,t=5}\ \varepsilon_{i,j})$	Monoculture + species interactions by composition	-1331	0.876	39.8	<0.0001

$a_{i,t=x}$ - monoculture yields of each species *i* at time x

$\beta_{t=0}\,(E,S)$ – slopes fitted between community yields and $\Sigma(a_{i,t=0}\ d_{i,j})$ at t=0 in each environment, E, and final richness level, S.

$\varepsilon_{i\ i,j}$ - the survival (1) or extinction (0) of each species in community *j* by the end

$b_{i,t=5}$ - monoculture yields of isolates of species *i* extracted from community *j* at t=5

C – a factor with levels denoting each community composition

Table S8. Simplified linear mixed effect model of community yields fitted with separate coefficients for each species in each environment and that vary over time. Note a full model including interaction terms with richness as well for all species could not be fitted. ANOVA of fixed effects is shown for conciseness.

Fixed effect	numDF	denDF	F-value	p-value
Intercept	1	194	4945.24	<.0001
log(richness)	1	185	132.631	<.0001
environment	2	185	391.819	<.0001
time	1	194	10.697	0.0013
spec.2	1	185	1.4	0.2383
spec.6	1	185	8.627	0.0037
spec.7	1	185	6.096	0.0145
spec.9	1	185	2.622	0.1071
spec.14	1	185	22.937	<.0001
spec.18	1	185	85.077	<.0001

spec.20	1	185	16.535	0.0001
spec.22	1	185	110.065	<.0001
spec.29	1	185	6.849	0.0096
spec.32	1	185	67.451	<.0001
spec.39	1	185	10.197	0.0017
spec.63	1	185	50.454	<.0001
log(richness):environment	2	185	15.605	<.0001
environment:time	2	194	11.807	<.0001
time:spec.2	1	194	0.176	0.6752
environment:spec.2	2	185	2.4	0.0936
time:spec.6	1	194	6.547	0.0113
environment:spec.6	2	185	5.771	0.0037
time:spec.7	1	194	0.574	0.4494
environment:spec.7	2	185	18.9	<.0001
time:spec.9	1	194	0.313	0.5766
time:spec.14	1	194	12.271	0.0006
environment:spec.14	2	185	6.644	0.0016
time:spec.18	1	194	7.612	0.0064
environment:spec.18	2	185	4.178	0.0168
time:spec.20	1	194	2.171	0.1423
environment:spec.20	2	185	11.149	<.0001
time:spec.22	1	194	3.579	0.06
environment:spec.22	2	185	9.313	0.0001
time:spec.29	1	194	2.251	0.1351
environment:spec.29	2	185	2.683	0.071
time:spec.32	1	194	2.429	0.1207
environment:spec.32	2	185	10.573	<.0001
time:spec.39	1	194	0.603	0.4385
environment:spec.39	2	185	1.692	0.1871
time:spec.63	1	194	2.501	0.1154
environment:spec.63	2	185	3.807	0.024
environment:time:spec.2	2	194	7.431	0.0008
environment:time:spec.6	2	194	2.974	0.0534

environment:time:spec.18	2	194	3.096	0.0475
environment:time:spec.20	2	194	12.614	<.0001
environment:time:spec.22	2	194	15.376	<.0001
environment:time:spec.29	2	194	1.884	0.1548
environment:time:spec.32	2	194	5.33	0.0056
environment:time:spec.39	2	194	2.416	0.0919

Table S9. Simplified linear mixed effect model of community yields in pH5 tea fitted with separate coefficients for each species that vary over time. Note a full model including interaction terms with richness as well for all species could not be fitted.

	Value	Std.Error	DF	t-value	p-value
Intercept	0.09678	0.01222	70	7.92	0
log(richness)	0.09204	0.02310	63	3.98	0.0002
time	-0.00003	0.00001	70	-4.08	0.0001
spec.2	-0.04386	0.01203	63	-3.65	0.0005
spec.6	-0.03777	0.01419	63	-2.66	0.0098
spec.7	0.03689	0.01219	63	3.03	0.0036
spec.18	0.03857	0.01297	63	2.97	0.0042
spec.20	-0.04237	0.01574	63	-2.69	0.0091
spec.22	0.01570	0.01362	63	1.15	0.2535
spec.29	-0.02056	0.01253	63	-1.64	0.1058
spec.32	-0.06178	0.01053	63	-5.87	0
spec.39	-0.01231	0.01376	63	-0.89	0.3744
spec.63	-0.06044	0.01131	63	-5.35	0
time:spec.6	0.00002	0.00001	70	1.64	0.1052
time:spec.20	0.00005	0.00001	70	4.76	0
time:spec.22	0.00004	0.00001	70	3.04	0.0033
time:spec.39	0.00002	0.00001	70	1.81	0.0747

Supporting Methods S1

16S sequencing

We amplified and sequenced the 16S rRNA gene of each isolate using the primers 8–27 (5’-AGA GTT TGA TCC TGG CTC AG-3’) and 1,512–1,492 (5’-ACG GTT ACC TTG TTA CGA CTT-3’). We added 0.6µM each primer for 25µl of PCR reaction using Illustra PureTaq Ready-To-Go PCR Beads (GE Healthcare, Little Chalfont, Buckinghamshire, UK). PCR conditions were 94ºC for 4 minutes, 30 cycles of 94ºC for 1 minute, 50ºC for 1 minutes and 72ºC for 2 min, with a final extension of 72ºC for 10 minutes.

Media preparation

Media prepared from beech and spruce leaves were supplemented with R2A agar ingredients: 0.375g proteose peptone, 0.375g casein hydrolysate, 0.375g yeast extract, 0.375g glucose, 0.374 g soluble starch, 0.225g dipotassium phosphate, 0.0375g magnesium sulphate, 0.225g sodium pyruvate per litre. Beech tea and spruce tea media initially had a pH of 7.1 and 6.8 respectively and were buffered to pH7 using 100mM phosphate buffer (5.8g/l monosodium phosphate and 8.2g/l disodium phosphate added). Beech tea for the pH5 treatment was buffered to pH5 by adding 13.6g/l monosodium phosphate and 0.2g/l disodium phosphate.

In some microcosms (those containing THB29 or THB39) a biofilm was sometimes present at the point of transfer. Before transferring to the fresh media and before reading the OD, we mixed the culture by pipetting up and down ~10 times. Samples were taken from half way between the surface and the bottom of the culture, and avoiding the walls of the bottle.

Supporting Results S1

One possible reason why extinction had little effect is that the species that had gone extinct by the end were already contributing little to community yields at the start. We tested this by refitting community yields at the start of the experiment to the sum of monoculture yields, but excluding species that were extinct by the end from the calculation and using final richness instead of starting richness. This measure indeed explained starting community yields better than the sum of all species present at the start (AIC=-936.5 versus 867.8). However, using this measure and the final richness for models led to the same conclusions regarding the roles of additive evolution versus evolution in interactions (table S7). Note that starting richness explained residual variation in these models (table S7), indicating that our starting treatments retained an effect, even though species richness declined over time.

107
